# Introduction to the *Toxins* Special Issue on Ergot Alkaloids

**DOI:** 10.3390/toxins7104232

**Published:** 2015-10-20

**Authors:** Christopher L. Schardl

**Affiliations:** Department of Plant Pathology, University of Kentucky, Lexington, KY 40546-0312, USA; E-Mail: schardl@uky.edu

Ergot alkaloids are among the most relevant natural products in the history of toxins and pharmaceuticals. Until the late 20th century, human and livestock exposure to ergot alkaloids was primarily through ingestion of “ergots,” which are spur-shaped or seed-like resting structures (sclerotia) of ergot fungi, the *Claviceps* species. Because ergots have similar density to grains, traditional threshing techniques generally failed to remove them, and outbreaks of ergot typically led to mass poisonings. The self-pollinating habit of wheat greatly limits ergot infestation, but other grain crops such as rye, barley and pearl millet more frequently host *Claviceps* ergots. Human poisoning came to be known in medieval Europe as “St. Anthony’s fire,” which was described as entailing a number of symptoms ranging from convulsions and hallucinations to dry gangrene and limb loss, and could be fatal. The causative agents of most ergot poisonings are the ergot alkaloid class of fungal metabolites, though some ergot fungi produce distantly related indole-diterpene alkaloids that are tremorgenic. Ergot alkaloids also have had medical applications for centuries, first in obstetrics to promote labor and reduce uterine hemorrhaging [1], and most recently in treatment of migraines and various neurological and endocrinological disorders including parkinsonism [2,3,4]. Their realized and hypothesized medicinal uses have encouraged intensive research since the 1950s culminating on the one hand in development of drugs both legal (e.g., bromocriptine) and illegal (e.g., lysergic acid diethylamide = LSD), and on the other hand in extensive knowledge of the enzymes, genetics, and diversity of ergot alkaloid biosynthetic pathways as described in this issue. Likewise, their presence in some endophyte-symbiotic grasses, and their negative effects on livestock production, have spurred research into the agroecology of these toxins. All of these subjects have advanced particularly far in recent years, so this is an opportune time to present this Special Issue on ergot alkaloids.

Nothing about ergot alkaloids is simple, considering their many neurotropic and pharmacological activities, the variety of ecosystems, plant-fungus infections and symbioses in which they are found, their effects on plant ecology and implications in livestock agriculture, their exceptional chemical diversity, and even the history and social implications of ergot alkaloid poisoning and recreational use of LSD. This Special Issue focuses on the ecological and agricultural aspects of the ergot alkaloids. An overview of the biosynthetic pathway is presented by Gerhards *et al.* [5], and Robinson and Panaccione [6] explore the biochemical basis for diversification of ergot alkaloids in nature as well as implications for laboratory manipulations of ergot alkaloid-producing fungi that result in profiles altered in sometimes unexpected ways. The genetics and genomic structures that underlie evolutionary diversification of these alkaloids are reviewed by Young *et al.* [7]. To aid the reader in cross-referencing these contributions, I present here a listing of the ergot alkaloid synthesis (*eas*) genes and the enzymes that they encode, as well as a key to the gene designations used for different organisms and on gene maps in this Special Issue (Table 1).

Considering that the many forms of ergot alkaloids have different pharmacological properties, providing for highly successful development of several important drugs, the basis for ergot-alkaloid diversity is of considerable interest. In recent years, it has become evident that several evolutionary processes have acted to diversify the array of ergot alkaloids produced by fungi. Differences in enzyme activities are evident at the levels of substrate specificity (LpsA), product specification (EasA, CloA) or both (EasG and possibly CloA) [6]. The “old yellow enzyme,” EasA, presents an outstanding example. This enzyme catalyzes reduction of the C8=C9 double-bond in chanoclavine I, but EasA isoforms differ in whether they subsequently catalyze reoxidation of C8–C9 after rotation. This difference distinguishes most Clavicipitaceae from Trichocomaceae, but in Clavicipitaceae it is also the key difference dividing the branch of classical ergot alkaloids from dihydroergot alkaloids, the latter often being preferred for pharmaceuticals due to their relatively few side effects.

Genomic alternations provide other means to alter and diversify ergot alkaloid profiles [7]. In most species examined the evidence indicates that *eas* genes occupy a single cluster, designated the *EAS* cluster. A set of seven orthologs is shared between Trichocomaceae and Clavicipitaceae, *dmaW*, *easF*, *easC*, *easE*, *easD*, *easA* and *easD*, listed in order of function of the enzymes that they specify for the biosynthesis of festuclavine (in Trichocomaceae) or agroclavine (in most Clavicipitaceae). Fungi in each family differ in subsequent modifications attributable mostly to enzyme classes common in specialized metabolite gene clusters, such as cytochromes P450 (*cloA*, *easK*, *easM*) and nonribosomal peptide synthetases (*lpsA*, *lpsB*, *lpsC*), as well as a prenyltransferase (*easL*) and acetyltransferase (*easN*). Thus, these clusters have been assembled by acquiring and neofunctionalizing genes. Relationships of the *lps* genes suggest that the process involved gene duplications, and, highlighting that possibility, the *Claviceps purpurea* 20.1 *EAS* cluster has duplicate *lpsA* genes that specify different ergopeptines. In fact, and quite mysteriously, *C. purpurea* lineages have evolved an exaltation of LpsA variants to give most of the 20 different ergopeptines found to date.

Evolutionary processes at the genome level sometimes lead to greater simplicity [7]. For example, some *Epichloë* species, as well as *Claviceps fusiformis*, have lost genes for late-pathway steps, and instead make the relatively simple clavines. Even the emergence of new late-pathway steps specified by *lpsC*, *easO* and *easP* leads to products such as ergonovine, LAH and ergine (lysergyl amide), which are much simpler in structure than ergopeptines. However, since these pathways seem inefficient at channeling to the final product (*i.e.*, “leaky”), more steps generally means a greater variety of ergot alkaloids, allowing the fungus to deal from an even deeper set of metabolites to defend itself and its host [6].

**Table 1:** Gene names and enzyme functions in ergot alkaloid biosynthesis.

General gene name	Abbreviation	Enzyme	Species and strain			
			*Neosartorya fumigatus* Af293	*Periglandula ipomoeae* IasaF13	*Claviceps purpurea* 20.1	*Epichloë festucae* Fl1
*easA*	*A*	chanoclavine-I aldehyde oxidoreductase; old yellow enzyme; reduction or isomerization of C8=C9 bond	*fgaOx3 (easA-red)*	*easA-iso*	*easA-iso*	*easA-iso*
*cloA*	*B*	agroclavine, festuclavine, elymoclavine or lysergol monooxygenase, oxidation of C17 to alcohol or carboxyl	–	*cloA*	*cloA*	*cloA*
*easC*	*C*	catalase involved in chanoclavine-I synthesis	*fgaCat*	*easC*	*easC*	*easC*
*easD*	*D*	chanoclavine-1 dehydrogenase	*fgaDH*	*easD*	*easD*	*easD*
*easE*	*E*	chanoclavine-I synthase	*fgaOx1*	*easE*	*ccsA*	*easE*
*easF*	*F*	dimethylallyltryptophan N-methyltransferase	*fgaMT*	*easF*	*easF*	*easF*
*easG*	*G*	agroclavine, festuclavine or pyroclavine synthase; reduction of C7=N iminium ion	*fgaFS*	*easG*	*easG*	*easG*
*easH*	*H*	ergopeptide lactam hydroxylase	–	*easH*	*easH*	*easH*
*easK*	*K*	cytochrome P450; possible festuclavine 9-monooxygenase	*fgaP450-1*	–	–	–
*easL*	*L*	fumigaclavine reverse prenyltransferase	*fgaPT1*	–	–	–
*easM*	*M*	cytochrome P450, possible festuclavine 9-monooxygenase	*fgaP450-2*	–	–	–
*easN*	*N*	fumigaclavine acetylase	*fgaAT*	–	–	–
*easO*	*O*	FAD-containing monooxygenase; putative ergonovine dehydrogenase	–	*easO*	–	–
*easP*	*P*	alpha/beta hydrolase fold protein; putative lysergic acid α-hydroxyethylamide (LAH) synthase	–	*easP*	–	–
*dmaW*	*W*	dimethylallyltryptophan synthase	*fgaPT2*	*dmaW*	*Cpd1*	*dmaW*
*lpsA*	*lpsA*	lysergyl peptide synthetase subunit 1; specifies amino acids 1, 2 and 3	–	*lpsA-ERB*	see *lpsA*1 and *lpsA*2 below	*lpsA-ERV*
*lpsA*1	*lpsA*1	lysergyl peptide synthetase subunit 1; specifies Ala, Phe and Pro	–	–	*Cpps1 = lpsA-ERA*	–
*lpsA*2	*lpsA*2	lysergyl peptide synthetase subunit 1; specifies Val, Leu and Pro	–	–	*Cpps4 = lpsA-ERK*	–
*lpsB*	*lpsB*	lysergyl peptide synthetase subunit 2; specifies lysergic acid	–	*lpsB*	*Cpps2*	*lpsB*
*lpsC*	*lpsC*	lysergyl peptide synthetase and reductase subunit; specifies L-alanine	–	*lpsC*	*Cpps3*	–

Additionally in this issue, Crews [8] provides details on chemical detection and analysis of ergot alkaloids by liquid chromatography and tandem mass spectrometry (LC-MS/MS), or by liquid chromatography with fluorescence detection (LC-FLD). Such sensitive and definitive approaches as currently available or under development are crucial to efforts in food and feed safety, studying ergot alkaloids in plant materials, and investigating activities and metabolism of ergot alkaloids in animals. 

Guerre [9] discusses the variety of ergot alkaloids produced by *Epichloë* species, which are notable for their ecological niches as systemic symbionts of cool-season grasses. Various toxic syndromes are associated with ergot alkaloids, commonly present in the grass, tall fescue (*Lolium arundinaceum*), with common strains of its endophyte, *Epichloë coenophiala*, as well as in perennial ryegrass (*Lolium perenne*) with common strains of *Epichloë festucae* var. *lolii*. Symptoms vary based on the chemical forms of the alkaloids, the additional presence of other alkaloids, such as indole-diterpenes, and environmental variables. In fact the lolitrems, a subclass of indole-diterpenes, are implicated in the most acute symptoms suffered by sheep and other livestock when grazed on perennial ryegrass. Whether there is any interaction between ergot alkaloids and indole-diterpenes has not been adequately addressed to date. 

A detailed overview of the toxicities in mammalian livestock is presented by Klotz [10]. Generally the activities are attributable to antagonism or agonism of neurotransmitters, including dopamine, serotonin and norepinephrine. The adrenergic blockage by ergopeptines (e.g., ergovaline or ergotamine) leads to potent and long-term vasoconstriction, and can result in reduced blood flow resulting in intense burning pain (St. Anthony’s fire), edema, cyanosis, dry gangrene and even loss of hooves in cattle or limbs in humans. Reduced prolactin due to ergot alkaloid activity on dopamine receptors in the pituitary is also common in livestock. Reduced serum prolactin is associated with various reproductive problems in cattle, and especially in horses, including agalactia and poor conception, and late-term losses of foals and sometimes mares due to dystocia and thickened placentas.

Miedaner [11] presents an excellent overview of ergot in Europe, including convulsive and gangrenous ergotism. He follows with descriptions of the life cycles of ergot fungi including *Claviceps africanus*, *Claviceps sorghi* and *C. purpurea*. Grass species with cleistogamous florets—*i.e.*, florets that self-pollinate before opening—tend to avoid ergot-fungus infections, and this largely explains why ergots are much more common on ears of rye and pearl millet than on wheat, barley and sorghum. Given the importance of cleistogamy as a protection from *Claviceps* spp., ergot is a problem during production of hybrids produced by employing male sterility or other means. Therefore, additional mechanisms of avoidance or resistance are of considerable interest. Miedaner [11] reviews the current knowledge of such mechanisms, and makes clear that more study is needed. Breeding and management practices are needed for hybrids that have full pollen production and fertility to reduce incidences of ergot.

This Special Issue also features several research papers, for which follow brief synopses. 

Steiner *et al.* [12] provide new insight into the interactions of morning glories with the epibiotic endophytes, the *Periglandula* species, which are responsible for occurrence of toxic levels of ergot alkaloids and, sometimes, indole-diterpenes in foliage and seeds of these plants. Peltate glandular trichomes on adaxial leaf surfaces secrete a variety of lipids, including several sesquiterpenes as well as palmitic acid. Given the physical associations of *Periglandula* mycelia with these glands, it seems likely that these lipids are the primary carbon and energy source for the fungus. Their ultrastructural studies reveal appressorium-like structures that are produced by *Periglandula* and associated with degradation of the cuticular layer of the gland surface. Furthermore, a UV-fluorescence study supported the previous observations by this group [13] that alkaloids were lost from fungal mycelia on leaf panels, but accumulated in the leaves. These results support a model for function of the glands in two-way transport, thus as a crucial interface enabling this symbiosis.

Dänicke [14] investigated ergot toxicity in Pekin ducks, and titrated the lowest observed adverse effect level (LOAEL) for total ergot alkaloids (TEA) at 0.6 mg TA/kg diet. Given that the current European Union threshold is 1 g ergot/kg unground cereal grains, and that the mean TEA for ergots was 0.7 mg/g, this pronounced sensitivity implied that the current regulations are insufficient to avoid toxicity to ducks. Within one week the choice-fed ducks learned to avoid ergot-containing diet, and the ducks dramatically decreased weight gain, though the ratio of feed intake to gain increased slightly but significantly in ducks fed increasing proportions of ergot in diets. 

The most famous—or infamous—of the Clavicipitaceae is *Claviceps purpurea*, which is the primary source of pharmaceutical ergot alkaloid, the cause of mass outbreaks of St. Anthony’s fire due to infection of rye and barley ears, and the species implicated rightly or wrongly in the Salem witch trials and others. The vast majority of ergopeptines are from strains identified as *C. purpurea* [7]. However, the circumscription of this species was recently refined in studies by Pažoutová *et al.* [15], in which *Claviceps humidiphila*, *Claviceps arundinis* and *Claviceps spartinae* were described. Negård *et al.* [16] have surveyed ergots in Norway and provide new insights into the taxonomic, chemotypic and ecotypic relationships of the *C. purpurea* complex. Their results support the recently proposed *C. purpurea* sensu stricto (s.s.), *C. humidiphila* and *C. arundinis*. Whereas ergopeptines were typical of *C. purpurea* s.s., *C. humidiphila* produced high levels of the ergopeptide lactams, ergocristam and ergosedmam. (Ergopeptide lactams are immediate precursors of ergopeptines, to which they can be converted by the action of the EasH hydroxylase [17].) Identification of indole-diterpenes from *C. arundinis* and *C. humidiphila* [16] is of interest in light of the defective indole-diterpene gene cluster in the sequenced genome of *C. purpurea* 20.1, and in keeping with the co-occurrence of indole-diterpenes and ergot alkaloids in other Clavicipitaceae [18].

It is my hope that this Special Issue will be a resource for many years to those scientists and lay persons interested in the agricultural and ecological relevance of ergot alkaloids, and their fascinatingly intricate biosynthetic pathways with enzymatic and evolutionary twists that have produced an extraordinarily diverse array of both problematic and useful compounds in this class.

## References

[1] De Groot A.N., van Dongen P.W., Vree T.B., Hekster Y.A., van Roosmalen J. (1998). Ergot alkaloids. Current status and review of clinical pharmacology and therapeutic use compared with other oxytocics in obstetrics and gynaecology. Drugs.

[2] Burn D. (2000). Neurology (2) Parkinson’s disease: Treatment. Pharm. J..

[3] Crosignani P.G. (2006). Current treatment issues in female hyperprolactinaemia. Eur. J. Obstet. Gynecol. Reprod. Biol..

[4] Micale V., Incognito T., Ignoto A., Rampello L., Sparta M., Drago F. (2006). Dopaminergic drugs may counteract behavioral and biochemical changes induced by models of brain injury. Eur. Neuropsychopharmacol..

[5] Gerhards N., Neubauer L., Tudzynski P., Li S.-M. (2014). Biosynthetic pathways of ergot alkaloids. Toxins.

[6] Robinson S.L., Panaccione D.G. (2015). Diversification of ergot alkaloids in natural and modified fungi. Toxins.

[7] Young C.A., Schardl C.L., Panaccione D.G., Florea S., Takach J.E., Charlton N.D., Moore N., Webb J.S., Jaromczyk J. (2015). Genetics, genomics and evolution of ergot alkaloid diversity. Toxins.

[8] Crews C. (2015). Analysis of ergot alkaloids. Toxins.

[9] Guerre P. (2015). Ergot alkaloids produced by endophytic fungi of the genus *Epichloë*. Toxins.

[10] Klotz J. (2015). Activities and effects of ergot alkaloids on livestock physiology and production. Toxins.

[11] Miedaner T., Geiger H.H. (2015). Biology, genetics, and management of ergot (*Claviceps* spp.) in rye, sorghum, and pearl millet. Toxins.

[12] Steiner U., Kucht S., Ahimsa-Müller M., Grundmann N., Li S.-M., Drewke C., Leistner E. (2015). The key role of peltate glandular trichomes in symbiota comprising clavicipitaceous fungi of the genus *Periglandula* and their host plants. Toxins.

[13] Markert A., Steffan N., Ploss K., Hellwig S., Steiner U., Drewke C., Li S.-M., Boland W., Leistner E. (2008). Biosynthesis and accumulation of ergoline alkaloids in a mutualistic association between *Ipomoea asarifolia* (Convolvulaceae) and a clavicipitalean fungus. Plant Physiol..

[14] Dänicke S. (2015). Ergot alkaloids in feed for Pekin Ducks: Toxic effects, metabolism and carry over into edible tissues. Toxins.

[15] Pažoutová S., Pešicová K., Chudíčková M., Šrůtka P., Kolařík M. (2015). Delimitation of cryptic species inside *Claviceps purpurea*. Fungal Biology.

[16] Negård M., Uhlig S., Kauserud H., Andersen T., Høiland K., Vrålstad T. (2015). Links between genetic groups, indole alkaloid profiles and ecology within the grass-parasitic *Claviceps purpurea* species complex. Toxins.

[17] Havemann J., Vogel D., Loll B., Keller U. (2014). Cyclolization of d-lysergic acid alkaloid peptides. Chem. Biol..

[18] Schardl C.L., Young C.A., Hesse U., Amyotte S.G., Andreeva K., Calie P.J., Fleetwood D.J., Haws D.C., Moore N., Oeser B. (2013). Plant-symbiotic fungi as chemical engineers: Multi-genome analysis of the Clavicipitaceae reveals dynamics of alkaloid loci. PLoS Genet..

